# Isolation and screening of wood-decaying fungi for lignocellulolytic enzyme production and bioremediation processes

**DOI:** 10.3389/ffunb.2024.1494182

**Published:** 2024-12-19

**Authors:** Anna Civzele, Linda Mezule

**Affiliations:** Water Systems and Biotechnology Institute, Faculty of Natural Sciences and Technology, Riga Technical University, Riga, Latvia

**Keywords:** biomass bioconversion, enzyme production, lignocellulolytic enzymes, textile dyes, wastewater treatment, wood-decaying fungi

## Abstract

The growing demand for novel enzyme producers to meet industrial and environmental needs has driven interest in lignocellulose-degrading fungi. In this study, lignocellulolytic enzyme production capabilities of environmental fungal isolates collected from boreal coniferous and nemoral summer green deciduous forests were investigated, using Congo Red, ABTS, and Azure B as indicators of cellulolytic and ligninolytic enzyme productions. Through qualitative and quantitative assays, the study aimed to identify promising species for lignocellulose-degrading enzyme secretion and assess their potential for biotechnological applications. Primary screening tests showed intensive enzyme secretion by certain isolates, particularly white rot fungi identified as *Trametes pubescens* and *Cerrena unicolor*. These fungi exhibited high efficiency in degrading Congo Red and Azure B. The isolates achieved up to a 93.30% decrease in Congo Red induced color intensity and over 78% decolorization of Azure B within 168 hours. Within 336 hours, these fungi reached nearly 99% removal of Congo Red and up to 99.79% decolorization of Azure B. Enzyme activity analysis confirmed the lignin-degrading capabilities of *T. pubescens*, which exhibited laccase activity exceeding 208 U/mL. Furthermore, *Fomitopsis pinicola* showed the highest cellulose-degrading potential among the studied fungi, achieving cellulase activity over 107 U/L during Congo Red decolorization. Previously undescribed enzyme-producing species, such as *Peniophora cinerea*, *Phacidium subcorticalis*, and *Cladosporium pseudocladosporioides*, also demonstrated promising lignocellulolytic enzyme production potential, achieving up to 98.65% and 99.80% decolorization of Congo Red and Azure B, respectively. The study demonstrates novel candidates for efficient lignocellulolytic enzyme production with broad biotechnological applications such as biomass conversion, wastewater treatment, textile dye and other complex chemical removal, and environmental remediation.

## 1 Introduction

Over the years, wood-decaying fungi have gained attention due to their remarkable capacity to break down complex plant materials into organic compounds with a wide spectrum of enzymes (Fen et al., 2014; Komorowicz et al., 2023). Fungi synthesize the enzymes to ensure lignocellulose degradation and facilitate the extraction of nutrients mandatory for their metabolism and growth (Sakekar et al., 2021). Among these enzymes, fungal cellulases ensure the hydrolysis of cellulose into simpler sugars (Behera et al., 2017; Singh et al., 2021), while certain strains of white rot fungi possess the ability to secrete ligninolytic enzymes, such as laccases and peroxidases, enabling them to effectively degrade complex lignin structures (Kumar and Chandra, 2020).

Due to the versatile catalytic activity, the potential of the lignocellulolytic enzymes extends beyond the lignocellulosic biomass degradation and can be applied in diverse biotechnological fields. For example, the ability of cellulolytic fungi to break down cellulose compounds into fermentable sugars holds the potential in biofuel production, including bioethanol (Fu et al., 2024). Cellulases are also utilized in the textile industry for fabric softening, biopolishing of textile fibers, and improving fabric quality (Kuhad et al., 2011; Singh et al., 2021). In the food and beverage industry, cellulases aid in the extraction and clarification of juices from fruits and vegetables, as well as in the brewing process for improving fermentation efficiency (Ejaz et al., 2021). Cellulolytic enzymes produced by lignocellulose-degrading fungi can also be involved in the paper industry for the modification of pulp properties and reducing energy consumption during paper production (Kuhad et al., 2011; Barrios et al., 2023). Moreover, cellulases may play a significant role in wastewater treatment and waste management to decompose cellulose-rich materials (Gautam et al., 2012; Bhardwaj et al., 2021).

Among the ligninolytic enzymes, laccases are known for their ability to oxidize various substrates, including phenolic compounds and dyes that have been recognized as persistent contaminants in wastewater treatment (Negi and Das, 2023). Laccases can also be used in lignin pretreatment (Atiwesh et al., 2021), improving lignocellulose degradation and bioenergy production processes. Peroxidases, the other group of ligninolytic enzymes, including manganese peroxidase (MnP), lignin peroxidase (LiP), and versatile peroxidases, are also involved in lignin degradation, with MnP exhibiting preference for Mn(II) as a substrate and LiP catalyzing the oxidation of non-phenolic lignin compounds (Conesa et al., 2002). Versatile peroxidases, on the other hand, exhibit a broader substrate specificity, making them adaptable to various industrial processes. Similarly to laccases, peroxidases have the potential for application in wastewater treatment, assisting in the removal of organic pollutants (Shanmugapriya et al., 2019). In the paper and pulp industry, MnPs and LiPs can be utilized for the delignification of wood pulp (Falade et al., 2016) and for bioleaching and bio-pulp purposes, providing an alternative to chemical and mechanical pulping (Fabbri et al., 2023). Moreover, fungal enzymes have a potential application in the food industry to enhance flavor, color, and texture of food products and improve their nutritional quality (Raveendran et al., 2018; Pouris et al., 2024).

The increasing need for new products and development of industrial and agro-food biotechnology has resulted in significant growth of the enzyme production market. Currently, fungal enzymes account for more than 50% of the total enzyme market (El-Gendi et al., 2021), with species from genera such as *Aspergillus*, *Trichoderma*, *Rhizopus*, and *Penicillium* dominating the commercial-scale production. However, the growing enzyme market has prompted a continuous search for new enzyme producing organisms with desirable industrial characteristics (El-Gendi et al., 2021) and strong price regulation of key market players (Custom Market Insights, 2024).

To facilitate the introduction of new ligninolytic enzyme producing fungi, a thorough screening of indigenous fungal isolates from temperate forests famous for their high fungal activity but rarely related as a source for novel enzyme-producing species was performed. To confirm the cellulolytic and ligninolytic enzyme production, quantitative measurements of enzymatic activity were performed in conditions mimicking lignocellulose biodegradation. This was achieved with the use of indicators for the detection of fungal enzyme secretion. Congo Red, 2,2’-azino-bis(3-ethylbenzothiazoline-6-sulfonic acid), known as ABTS, and Azure B were selected for hydrolyzing and oxidative enzyme activity assessment. Congo Red is a synthetic dye commonly used for screening of fungal cellulolytic enzyme production (Carder, 1986). In the presence of these enzymes, the initial red coloration changes to orange or yellow. The observable color change serves as a qualitative indicator of cellulase activity, allowing for the screening and identification of microorganisms capable of cellulose degradation. ABTS is a non-phenolic dye that undergoes oxidation in the presence of laccases. The release of these enzymes results in the formation of the blue-green or purple color, which serves as an indicator of laccase activity (More et al., 2011). Azure B is a triarylmethane dye with a structural resemblance to lignin, commonly used as an indicator for ligninolytic enzyme activity. The decolorization of Azure B signifies the release of laccase and peroxidases, such as LiP and MnP, produced by fungi (Yang et al., 2021). Therefore, by evaluating and comparing the isolate enzymatic activity on various substrates, this study demonstrates novel candidates capable of producing highly active lignocellulolytic enzymes with potential industrial benefit, biotechnological application, and bioremediation potential, particularly in dye degradation.

## 2 Materials and methods

### 2.1 Fungi and culture conditions

Twenty fungal strains showing wood – decaying abilities were collected from Latvian mixed forests (boreal coniferous and nemoral summer green deciduous forests; 56.940054, 24.410492) and isolated in September 2023. Samples were collected from fallen birch and pine trees using a sterile knife and stored in sealed plastic bags to avoid contamination. The isolation of harvested fungal species was performed by cultivating the collected samples on Potato Dextrose Agar (PDA) (Oxoid Ltd., Basingstoke, Hants, UK) at 25°C and 80% relative humidity (rH) in a climate chamber (KBF 115, BINDER GmbH, Tuttlingen, Germany). After incubating for 7 days, mycelial disks from the grown isolate samples were transferred onto fresh PDA plates to establish monocultures of the harvested fungi through repeated cultivation.

The selected cultures of isolated fungal strains, demonstrating enzyme production potential, were sent for morphological and DNA-sequence based identification (ITS and D1/D2 LSU) to Belgian Coordinated Collections of Microorganisms, Agro-food & Environmental Fungal Collection (UCLouvain, Belgium).

Additionally, to evaluate the lignocellulolytic enzyme production capabilities of environmental isolates against established, well-characterized strains of white rot fungi, commercially available cultures of *Irpex lacteus* DSM 9595, *Pleurotus dryinus* (Pers.) *P. Kumm*, *Pleurotus ostreatus* DSM 1020, *Bjerkandera adusta* DSM 23426, *Trametes versicolor* DSM 6401, *Pycnoporus cinnabarinus* (Fr.) *P. Karst*, *Phanerochaete chrysosporium* DSM 9620, and *Trichoderma reesei* DSM 768 were used in this study. All fungal strains were maintained on PDA medium at 2–6°C.

### 2.2 Experimental conditions for qualitative screening tests

To prepare the agar medium for the screening tests, 0.8 g KH_2_PO_4_, 0.4 g K_2_HPO_4_, 0.5 g MgSO_4_·7H_2_O, 2 g NH_4_NO_3_, 2 g yeast extract, 10 g glucose, and 15 g agar (pH 5.5 ± 0.2) were added per L of distilled water. The prepared agar media were sterilized by autoclaving at 121°C for 15 min.

To detect the ability of selected fungal strains to secrete cellulolytic enzymes, agar media was supplemented with 0.5% (w/v) carboxymethylcellulose sodium salt (CMC) and 0.1% (w/v) Congo Red. For the determination of the ligninolytic enzyme production, 0.1% (w/v) ABTS (2,2′-Azino-bis(3-ethylbonzotiazoline-6-sulfonic acid)) diammonium salt) and 0.01% (w/v) Azure B were used as reaction substrates and supplemented to the agar media. The agar media with no indicator substrates was used as a control.

After the agar media preparation, ~1 cm^2^ mycelial disk of each selected fungal species was placed on each type of agar, and the agar plates were incubated for 336 hours at 25°C and 80% rH in a constant climate chamber (KBF 115, BINDER GmbH, Tuttlingen, Germany). Visual inspection of the agar plates and oxidation formation were performed daily to determine the qualitative changes. For the quantitative analysis, the percentage of the agar plate area that was covered by fungal mycelium and underwent substrate oxidation was determined daily by measuring the diameter of the fungal mycelium and the formed oxidation zone. All experiments and measurements were performed in 3 independent repetitions.

### 2.3 Experimental conditions for quantitative tests

To determine the cellulolytic and ligninolytic enzyme secretion, liquid medium with the same proportions of chemicals, excluding agar, was supplemented either with Congo Red or Azure B and inserted into 250 mL Erlenmeyer flasks. The composition of the media was thoroughly mixed and autoclaved at 121°C for 15 minutes.

Pieces of mycelium cut from approximately 1 cm x 2 cm segments of each fungal culture selected for the quantitative study based on the qualitative screening results were inoculated in the previously described types of liquid broth and incubated for 336 hours at 30°C and 150 rpm in an orbital shaker (New Brunswick™ Innova^®^ 43, Eppendorf Austria GmbH, Wien, Austria). The samples of liquid media were daily aseptically collected for the analysis. All experiments and measurements were performed in 3 independent repetitions. Flasks containing Congo Red and Azure B medium without fungi and inactivated fungi were used as the controls. For the experiments with inactivated fungi, the cultures were inactivated by autoclaving for 30 minutes at 121°C.

Daily samples during quantitative experiments were analyzed by measuring the absorption using a microplate reader (The CLARIOstar^®^ Plus, BMG Labtech, Germany) at specific wavelengths: 500 nm for Congo Red and 640 nm for Azure B supplemented liquid media samples against the time zero reading. The selection of these wavelengths was experimentally determined to maximize sensitivity to the absorption changes induced by the respective dyes.

To analyze the decolorization of Congo Red and Azure B-containing medium, the percentage of the color intensity was calculated using Equation 1, where Abs_0_ is the initial absorbance of each medium supplemented with dye, while Abs_N_ is the absorbance of the liquid medium after fungal treatment.


(1) $$\text{Decolorization}\;(\%)\;=\;\frac{{\text{Abs}}_{0}-{\text{Abs}}_{\text{N}}}{{\text{Abs}}_{0}}\;\times \;100\%$$

### 2.4 Enzyme activity measurements

Lignocellulose-degrading enzyme activity was assessed in samples collected during the quantitative experiments. The samples were centrifuged at 8500 rcf for 5 minutes and the supernatant was then used in the assessment.

To evaluate cellulose-degrading enzyme activity, fungal cellulase activity was analyzed according to the colorimetric assay described in the Mangan et al. (2016) study. The cellulase activity was determined by incubating the supernatant of the sample with a substrate solution containing β-glucosidase at 40°C for 10 minutes. After addition of the stopping reagent, 2% w/v Tris solution (pH 9), the absorbance of the resulting mixture was determined using a microplate reader (The CLARIOstar^®^ Plus, BMG Labtech, Germany) at 400 nm against the time zero reading for the respective substrate.

For ligninolytic enzyme activity, fungal laccase activity was determined by the ABTS oxidation method (Wolfenden and Willson, 1982). The reaction mixture contained 10 mM ABTS, 0.1 M sodium acetate buffer (pH 4.5), and the supernatant of the collected sample. Absorbance was measured using a microplate reader (The CLARIOstar^®^ Plus, BMG Labtech, Germany) at 436 nm against the distilled water as a blank.

### 2.5 Statistical analysis

For statistical analysis, t-test and ANOVA was performed in Microsoft Excel (significance level ≤ 0.05).

## 3 Results

### 3.1 Identification and screening of wood-decaying fungi

To identify lignocellulolytic enzyme production abilities, preliminary screening tests were conducted on twenty fungal isolates harvested in mixed forests along with eight commercial white rot fungi. During screening on solid media, ten of the previously isolated fungi did not exhibit any signs of enzyme production, showing no visible effects on any of the dye-supplemented agar media, and were not further identified. However, the remaining ten isolates demonstrated diverse cellulolytic and ligninolytic enzyme secretion capacity. The species of these fungi were subsequently identified as *Fomitopsis pinicola* (Swartz) P. Karsten, *Trichoderma atrobrunneum*, *Trichoderma paraviridescens*, *Fusarium graminearum*, *Cerrena unicolor* (Bulliard) Murrill, *Trametes pubescens* (Schumacher) Pilát, *Peniophora cinerea* (Pers.) Cooke, *Phacidium subcorticalis*, *Trichoderma polysporum* (Fries) Rifai, and *Cladosporium pseudocladosporioides* Bensch.

**Figure 1:**
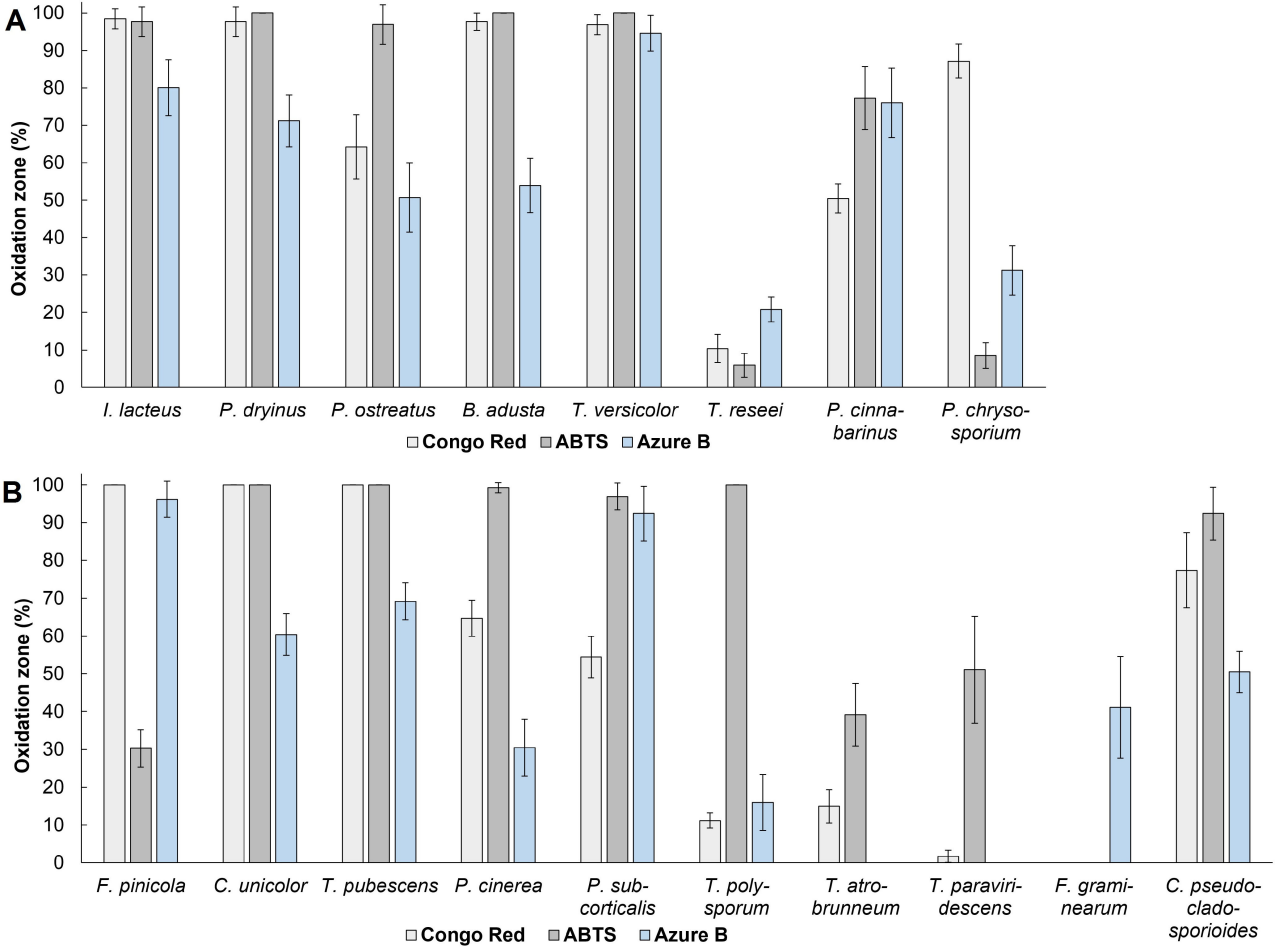
The percentage of oxidation zone formed from the total agar area after 336 hours of cultivating **(A)** fungal commercial strains and **(B)** wood-decaying environmental isolates. Standard deviation represents the average value from three independent repeats.

During the qualitative screening tests, *C. unicolor*, *T. pubescens*, and *P. cinerea* exhibited the most intense oxidation of ABTS, resulting in dark green to dark purple color formation in 99.22% to 100% of the agar media area, highlighting their ability to produce laccases (**Figure 1**). *C. unicolor* and *T. pubescens* also affected 100% of the solid media area supplemented by Congo Red, displaying intense cellulolytic enzyme secretion. *F. pinicola* also caused 100% decolorization of Congo Red media and was able to fade 96.15 ± 4.77% of the Azure B-containing agar area, demonstrating potential production capability of cellulases and peroxidases. The decolorization of Azure B was also achieved in the presence of *P. subcorticalis*, *T. pubescens*, *C. unicolor*, and *C. pseudocladosporioides*, demonstrating their ability to produce ligninolytic enzymes. *C. pseudocladosporioides*, *P. subcorticalis*, along with *T. polysporum*, also exhibited significant oxidation of ABTS, with 92.39 ± 6.94%, 96.91 ± 3.57%, and 100% color formation on ABTS-agar media, respectively. However, these isolates displayed a lower impact on Congo Red, *as C. pseudocladosporioides* affected 77.39 ± 10.01% of Congo Red solid media, *P. subcorticalis* affected 54.44 ± 5.50%, and *T. polysporum* oxidized only 11.17 ± 1.99% of the agar media, indicating less pronounced signs of cellulolytic enzyme activity.

**Figure 2:**
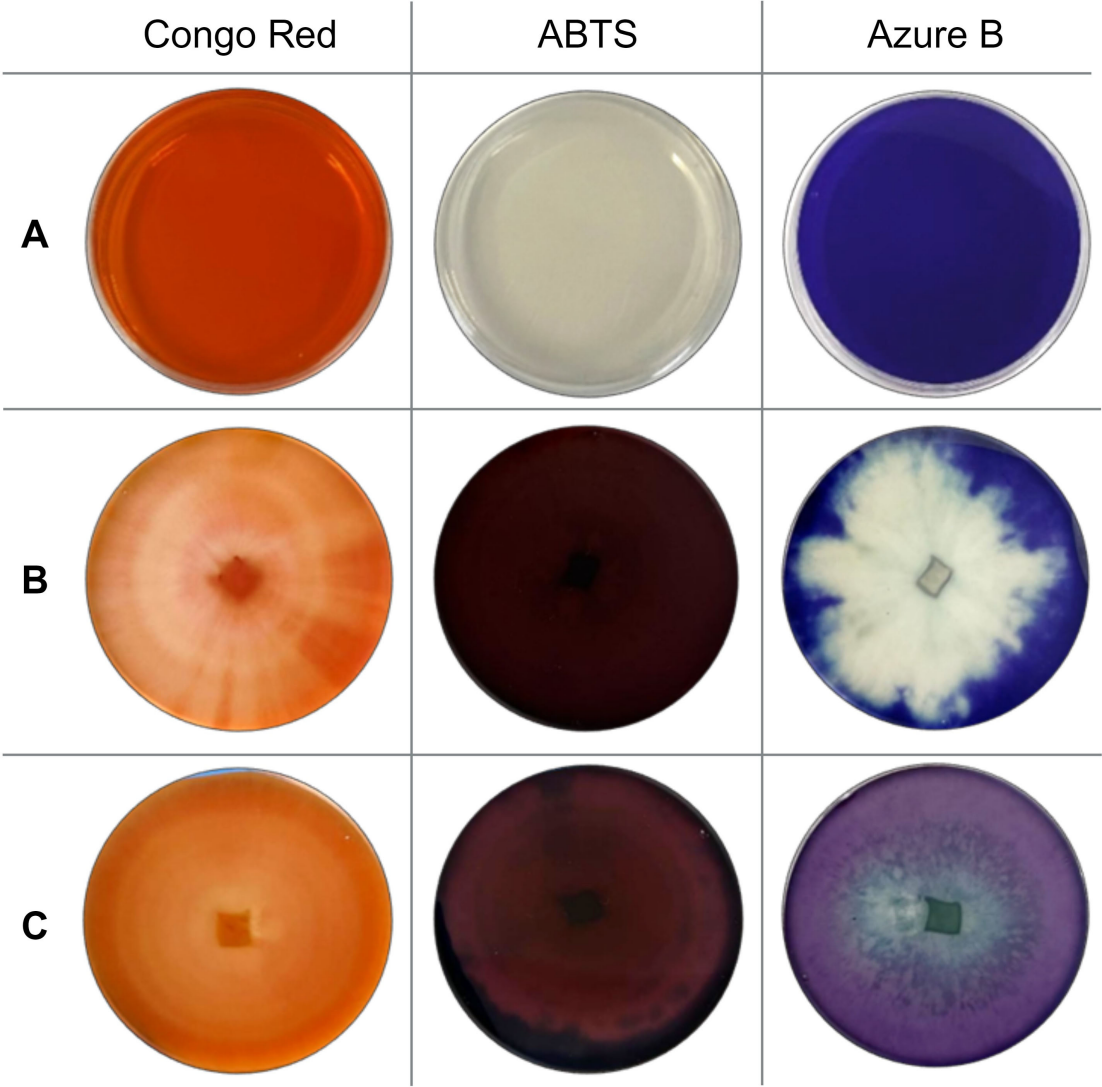
Screening results **(A)** before cultivation (control); **(B)** after 168 hours of *T. pubescens* cultivation; and **(C)** after 168 hours of *T. versicolor* cultivation.

Based on the screening results, the most potent fungal isolate to produce lignocellulolytic enzymes was *T. pubescens*. In the presence of this fungus, 100% of Congo Red and ABTS-containing solid medium area was affected. Additionally, intense oxidation of Azure B agar occurred: 69.57 ± 4.90% of the agar medium area was completely decolorized after 168 hours of incubation (**Figure 2**).

Among the commercial strains studied, five white rot fungi – *I. lacteus*, *P. dryinus*, *P. ostreatus*, *B. adusta*, and *T. versicolor* exhibited rapid and intense oxidation of ABTS. After 336 hours of incubation, these fungi oxidized 96.93 – 100% of the solid medium area (**Figure 1**). When cultivated on Congo Red-containing agar, the most effective decolorization was observed for *I. lacteus*, *P. dryinus*, *B. adusta*, *T. versicolor*, and *P. chrysosporium*, where 87.20 – 98.44% of the medium area was decolorized from bright red to yellow, suggesting the secretion of cellulolytic enzymes. The most rapid and intensive decolorization of Azure B, thus, the ability to produce ligninolytic enzymes, was observed during the growth of *T. versicolor*. The enzymatic activity of this fungi affected almost 95% of the solid medium with Azure B, leading to a transition from dark blue to light purple color (**Figure 2**). This white rot fungus also exhibited the highest potential of lignocellulolytic enzyme production during the screening study, oxidizing 96.90 ± 2.68% of Congo Red, 100% of ABTS, and 94.62 ± 4.78% of Azure B-containing solid medium within 336 hours.

Based on the qualitative screening results, fungal cultures demonstrating both cellulolytic and ligninolytic enzyme production ability were chosen for further enzyme production experiments. Thereby, 6 commercial strain white rot fungi and 6 fungal species isolated from the environment were used in the tests with Congo Red and Azure B-containing liquid media.

### 3.2 Determination of enzyme production potential in liquid media

#### 3.2.1 Decolorization of Congo Red

**Figure 3:**
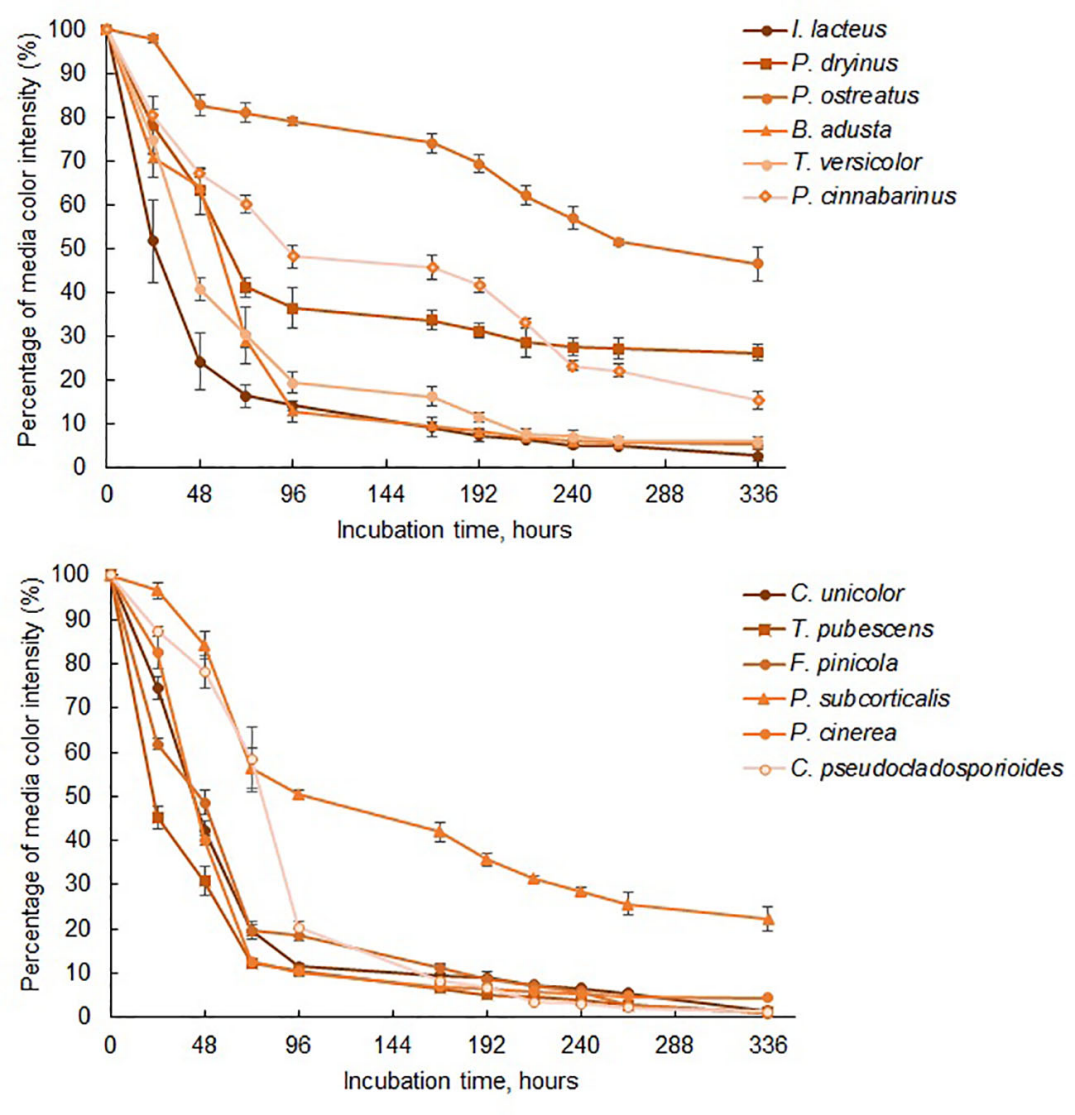
The reduction of color intensity of Congo Red-containing media during 336 hours of fungal cultivation. Standard deviation represents the average value from three independent repeats.

Among the commercially obtained strains, *I. lacteus* demonstrated the most rapid and efficient decolorization of Congo Red. The color intensity of the respective medium decreased by nearly 50% within the first 24 hours, over 75% after 48 hours, and reached 83.62 ± 2.58% reduction after 72 hours (**Figure 3**). After 336 hours of incubation, 97.36 ± 1.28% decolorization of Congo Red was observed. Similar decolorization efficiency was also observed when cultivating *B. adusta* and *T. versicolor*, which reduced the color intensity by 94.54 ± 0.48% and 94.10 ± 1.12%, respectively, within the same timeframe. However, the decolorization rate of these fungi in the first 72 hours was significantly lower (*p* < 0.05) when compared to *I. lacteus*: 0.98% per hour and 0.97% per hour against 1.16% per hour, respectively. Nevertheless, from 192 to 336 hours of incubation, the decolorization efficiency of all three fungi did not significantly differ (*p* > 0.05).

Lower efficiency of color removal was observed in *P. dryinus* and *P. cinnabarinus*. Despite this, the results still highlight the ability of these fungi to secrete cellulolytic enzymes, based on the observed reduction in the color intensity of Congo Red media by 73.20 ± 1.87% and 84.58 ± 2.04%, respectively. The lowest decolorization efficiency among the commercially obtained fungi was detected in *P. ostreatus*. This fungus also demonstrated the lowest degradation rate of Congo Red within the first 72 hours of the incubation (0.26% per hour) among the commercial and isolated fungal species. This aligns with the qualitative screening results, where *P. ostreatus* exhibited a lower effect on Congo Red medium (64.19 ± 8.53%) compared to *I. lacteus*, *P. dryinus*, *B. adusta*, and *T. versicolor*. In the liquid media, this fungus displayed 53.59 ± 3.76% decolorization efficiency after 336 hours, and the highest dye degradation rate was 0.63% per hour observed between 24 and 48 hours of incubation. The average rate of Congo dye decolorization by this fungus was 0.15% per hour.

**Figure 4:**
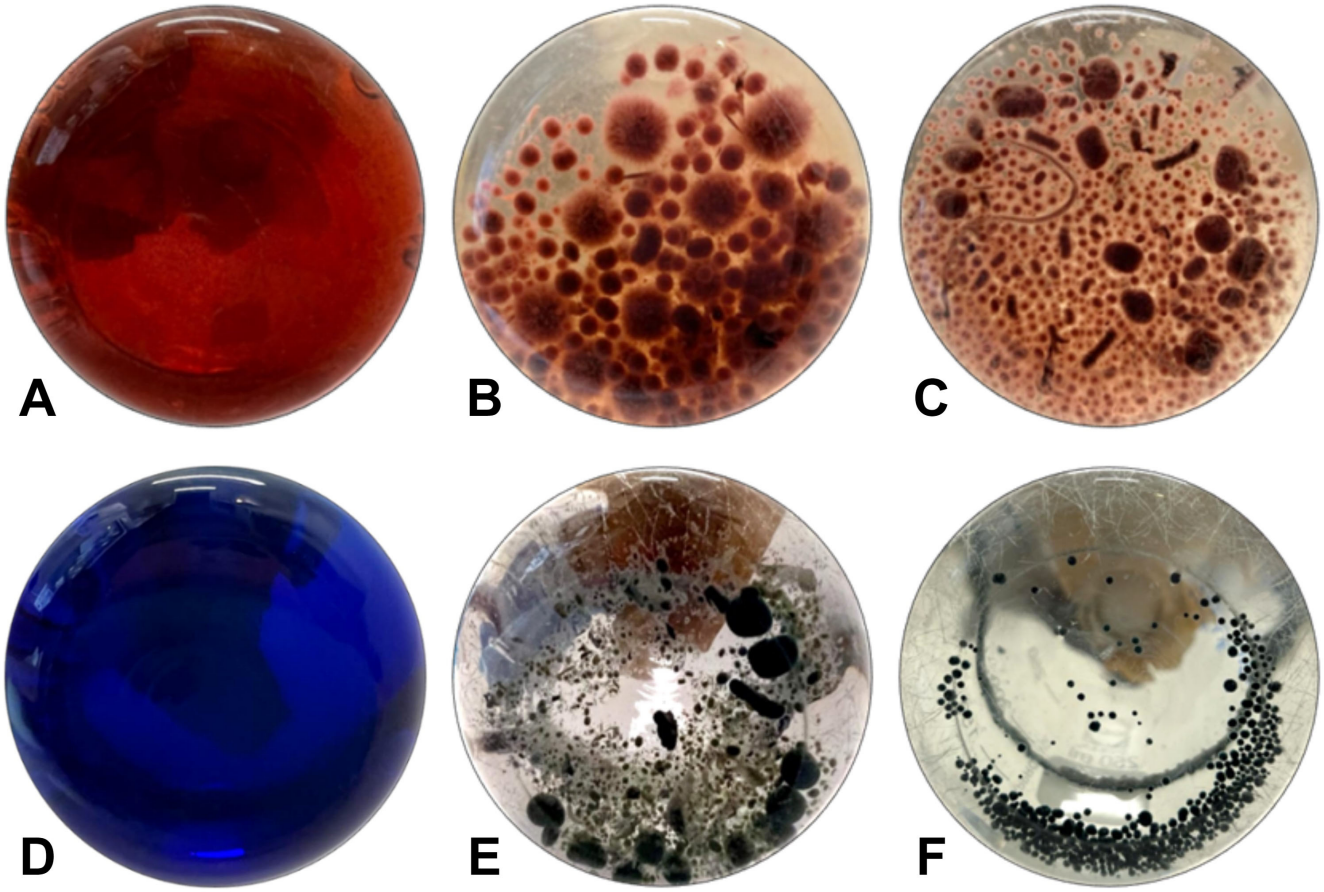
Decolorization of **(A)** Congo Red by **(B)** *I. lacteus* and **(C)** *F. pinicola* and decolorization of **(D)** Azure B by **(E)** *B. adusta* and **(F)** *C. pseudocladosporioides* after 336 hours of incubation.

While all fungi reduced the color intensity of the liquid media, indicating the potential presence of cellulase-degrading enzymes, *I. lacteus, B. adusta*, and *T. versicolor* can be characterized as the most efficient cellulolytic species among the studied commercially obtained fungi. The highest cellulase activity was detected in *I. lacteus* (up to 16.92 ± 2.01 Units/L after 48 hours of incubation), which also demonstrated the most efficient decolorization of Congo Red media. Similarly, *T. versicolor* exhibited its highest cellulolytic enzyme activity after 48 hours of cultivation in Congo Red-containing media, achieving 8.40 ± 3.19 Units/L. In. contrast, *B. adusta* reached its peak cellulolytic activity in 72 hours, exhibiting cellulase activity of 5.75 ± 1.76 Units/L. The activity of other commercial fungi was significantly lower (*p* < 0.05) and remained under 2 Units/L throughout the incubation in Congo Red media. In comparison to fungi from culture collections, the fungal isolates exhibited significantly higher Congo Red decolorization efficiency (*p* < 0.05), indicating a higher potential for cellulolytic enzyme production. Except for *P. subcorticalis*, all fungi demonstrated over an 86% decrease in color intensity within 168 hours of incubation. Among the previously described fungi, only *I. lacteus* and *B. adusta* were able to achieve similar efficiency. After 336 hours, more than 95% decolorization efficiency was achieved by *T. pubescens*, *F. pinicola*, *C. unicolor*, *P. cinerea*, and *C. pseudocladosporioides.* Notably, *F. pinicola* exhibited the highest efficiency, reducing the color intensity by 99.06 ± 0.11%, nearly achieving complete decolorization of Congo Red (**Figure 4**). In comparison to *I. lacteus* that showed to be the most efficient fungus obtained from culture collection, *C. unicolor* (98.57 ± 0.34%), *C. pseudocladosporioides* (98.66 ± 0.14%), and *T. pubescens* (98.91 ± 0.23%) had high removal efficiencies after 336 hours. The environmental isolates also demonstrated higher Congo Red decolorization rates in the first 96 hours, where the highest observed rates were 1.23 ± 0.01% per hour in *T. pubescens* and 1.22 ± 0.01% per hour in *P. cinerea* samples. At the same time, among the commercially obtained cultures, the highest decolorization rate in the early stage of the incubation was observed in *I. lacteus*, which ensured the decrease in the color intensity by 1.16% per hour. High rates of Congo red decolorization demonstrated by the isolated fungi underscore the efficiency of *T. pubescens* and *P. cinerea* as potent alternatives to *I. lacteus* in environmental applications, showing their potential for rapid dye degradation.

Furthermore, when compared to commercial strains, the cellulolytic activity analysis revealed significantly higher activity in environmental isolates (*p* < 0.05). The highest enzymatic activity was detected in *F. pinicola*. This brown rot fungus exhibited cellulase activity of 79.56 ± 8.23 Units/L within the first 24 hours, reaching its peak – 107.32 ± 12.21 Units/L, after 72 hours of cultivation in Congo Red-containing media. In contrast, cellulolytic activity comparable to the commercial fungi was observed in *T. pubescens* and *C. unicolor*, with maximum cellulase activities of 18.34 ± 2.90 Units/L and 10.30 ± 1.38 Units/L, respectively, after 72 hours. The highest enzymatic activity shown by *C. pseudocladosporioides* – 9.68 ± 2.30 Units/L, was observed already within 24 hours, followed by a gradual decrease to 2.90 ± 0.32 Units/L after 168 hours. *P. cinerea* exhibited similar cellulolytic activity to *B. adusta*, reaching the peak of 5.52 ± 2.46 Units/L after 72 hours of incubation. The cellulolytic activity of *P. subcorticalis* remained below 2 Units/L throughout the incubation in Congo Red-containing media.

Notably, the results and the high decolorization efficiency were solely observed in the presence of active fungal cultures, as inactivated fungi displayed no significant changes in Congo Red-induced coloration throughout the entire experimental duration (*p* > 0.05). The highest decolorization efficiency achieved by inactivated fungi was 7.91 ± 5.36%, observed during cultivation of *I. lacteus*, while the other examined fungi exhibited less than 5% decolorization of Congo Red-induced color within 336 hours. This observation demonstrates the crucial role of active fungi in Congo Red decolorization through cellulolytic enzyme activity and active fungal metabolism, rather than the physical adsorption process for effective dye degradation.

#### 3.2.2 Decolorization of Azure B

**Figure 5:**
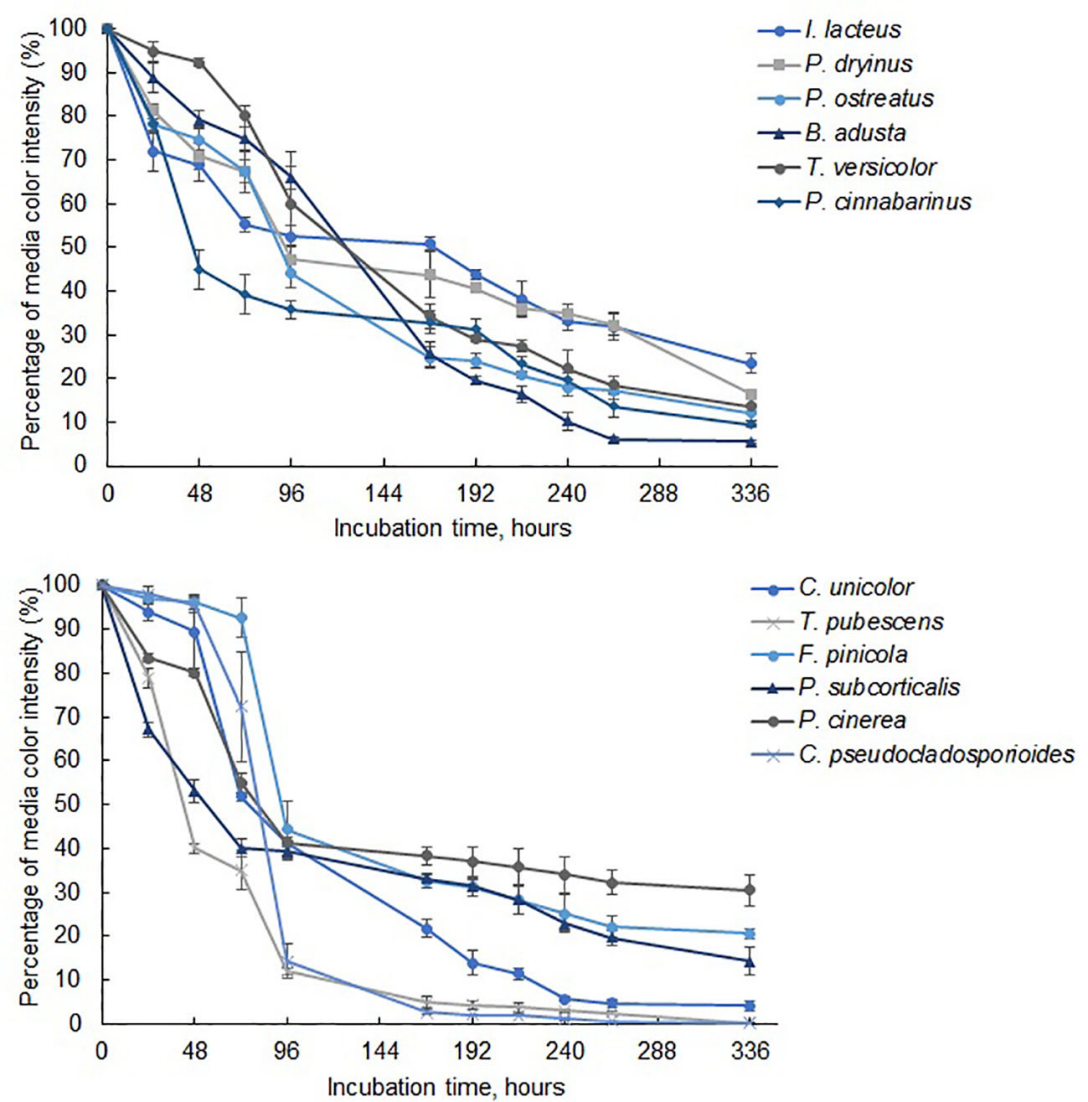
The reduction of color intensity in media containing Azure B during 336 hours of fungal cultivation. Standard deviation represents the average value from three independent repeats.

The most efficient Azure B degradation by commercially available fungi was ensured by *B. adusta*, which reduced the color intensity by 93.93 ± 0.55% within 264 hours (**Figure 5**) with the average decolorization rate of 0.36% per hour. After 264 hours, Azure B removal efficiency did not change significantly (*p* > 0.05), and the decolorization rate reduced to 0.007% per hour. More than 90% Azure B decolorization efficiency within 336 hours was also achieved by *P. cinnabarinus*. This white rot fungus also displayed the most rapid dye removal within the first 96 hours, and the Azure B decolorization rate of 1.15 ± 0.09% per hour was observed after 48 hours of incubation. After 336 hours, slightly lower decolorization efficiency was observed when cultivating *P. ostreatus*, *T. versicolor*, and *P. dryinus*, which ensured 87.84 ± 3.02%, 86.40 ± 3.10%, and 83.44 ± 0.47% decrease in Azure B color intensity. However, despite the lower efficiency after 336 hours than *B. adusta*, these fungi demonstrated high rates of Azure B decolorization within the early stage of the incubation – 0.58% per hour, 0.42% per hour, and 0.55% per hour, respectively. When compared with other white rot fungi, *I. lacteus*, showed the lowest efficiency reducing the color intensity of Azure B by 76.58 ± 2.32% after 336 hours. Nevertheless, all commercially obtained fungal strains ensured more than 50% decolorization of Azure B within 168 hours and more than 75% in 336 hours, indicating on apparent presence of ligninolytic enzymes.

To detect the ligninolytic enzyme-producing potential of the studied fungi, laccase activity was also analyzed as a representative enzyme. In the commercial fungal cultures, *B. adusta*, which demonstrated the most efficient decolorization of Azure B, exhibited a maximum laccase activity of 5,901.42 ± 1,585.13 Units/L (5.90 ± 1.59 U/mL) after 72 hours of incubation. Despite its lower Azure B decolorization efficiency, *T. versicolor* and *P. ostreatus* were determined to be significantly more active laccase producers (*p* < 0.05). These fungi achieved laccase activity levels of 24.48 ± 7.04 U/mL and 15.37 ± 1.14 U/mL, respectively. *P. dryinus* exhibited its highest laccase activity of 3.54 ± 1.69 U/mL during the incubation period, reaching this level after 96 hours. In contrast, the laccase activity of *I. lacteus* and *P. cinnabarinus* samples was significantly lower (*p* < 0.05) and remained below 1 U/mL throughout the incubation period.

Among the identified fungal isolates, the highest decolorization efficiency of Azure B was observed in *T. pubescens* and *C. pseudocladosporioides*, which ensured 99.79 ± 0.09% and 99.78 ± 0.11% removal of Azure B from the liquid media within 336 hours. The most rapid decrease in the color intensity of Azure B in samples with *T. pubescens* was observed at 48-hour incubation, when the dye decolorization rate was 1.25% per hour, achieving 60.00 ± 1.01% Azure B degradation. In the *C. pseudocladosporioides* sample (**Figure 4**), the most significant decolorization occurred from 48 to 96 hours, where the color intensity was reduced by 81.16% with a 1.70% per hour decolorization rate, achieving 85.64 ± 3.80% dye removal after 96 hours. Therefore, these fungal strains ensured more than 95% decrease in the color intensity within the first 168 hours. Afterwards, the dye degradation rate did not differ significantly between these two fungi (*p* > 0.05) and was reduced to 0.028% per hour r in *T. pubescens* and 0.015% per hour in *C. pseudocladosporioides*.

The decrease in the color intensity for more than 90% was observed during the cultivation of *C. unicolor*. This fungal strain ensured 94.28 ± 0.64% within 240 hours, reaching 95.83 ± 1.04% in 336 hours of incubation. However, when compared to *T. pubescens* and *C. pseudocladosporioides*, the decolorization efficiency of *C. unicolor* within 216 hours was significantly lower (*p* < 0.05).

Despite slightly lower efficiency, *P. subcorticalis, F. pinicola*, and *P. cinerrea* also showed the potential of ligninolytic enzyme secretion, ensuring the decolorization of Azure B by 85.66 ± 3.10%, 79.44 ± 1.21%, and 69.52 ± 3.44% within 336 hours, respectively. At the same time, inactivated fungal samples showed no significant changes (*p* > 0.05) in Azure B-containing media color intensity.

The highest decolorization rate in samples with added *P. subcorticalis* was observed within the first 48 hours, when the supplemented dye was decolorized by 0.98% per hour. However, after 48 hours, the decolorization rate of this fungus decreased significantly to 0.09% per hour. In the first 72 hours, the lowest efficiency in Azure B degradation was observed in *F. pinicola*, where the color intensity was reduced only by 7.32 ± 4.32%. However, from 72 to 96 hours of incubation, the decolorization rate rapidly increased to 2.02% per hour, reducing the Azure B-induced coloration by 55.71 ± 5.50% and becoming steady after 96 hours at 0.10% per hour. Overall, all natural isolates ensured more than 70% reduction in Azure B within 336 hours.

When compared to commercial fungal strains, some environmental isolates demonstrated similar or even higher laccase activity during cultivation in Azure B-containing media. Among them, *T. pubescens* exhibited the highest laccase production, with activity levels significantly exceeding the results observed in other commercial and environmental fungal strains (*p* < 0.05). This fungus achieved the laccase activity of 181,504.51 ± 20,734.12 Units/L (181.50 ± 20.73 U/mL) within the first 24 hours, reaching its peak activity of 208,595.50 ± 6,683.88 Units/L (208.60 ± 6.68 U/mL) after 72 hours. Despite significantly lower activity compared to *T. pubescens* (*p* < 0.05), *C. unicolor* exhibited laccase activity comparable to the levels observed in commercial *T. versicolor*. The peak activity of *C. unicolor* was 23,235.54 ± 1,971.99 Units/L (23.24 ± 1.97 U/mL) observed after 72 hours. Although *P. cinerea* showed the lowest decolorization efficiency among the environmental isolates, it demonstrated notable potential of active lignin-degrading enzyme production, with the observed laccase activity of 7,453.88 ± 1,804.31 Units/L (7.45 ± 1.80 U/mL), significantly higher (*p* < 0.05) than levels observed in the commercial strains *P. dryinus*, *I. lacteus*, and *P. cinnabarinus*. *C. pseudocladosporioides* exhibited its highest laccase activity of 2,231.02 ± 903.17 Units/L (2.23 ± 0.93 U/mL) at 48 hours. Meanwhile, the activity of *P. subcorticalis* and *F. pinicola* was significantly lower (*p* < 0.05) and remained below 1 U/mL throughout the incubation in Azure B media.

## 4 Discussion

This work has found a substantial richness in terms of lignocellulolytic enzyme-producing fungi at the boreal coniferous and nemoral summer green deciduous forests. Six isolates exhibited signs of pronounced enzyme-producing capability. Among these isolates, one species was identified as *F. pinicola*, a brown rot fungus known for its capacity to degrade cellulose and hemicellulose components of lignocellulosic materials while only slightly modifying lignin (Andlar et al., 2018; Qi et al., 2023). Additionally, three samples were identified as white rot fungi: *C. unicolor*, *T. pubescens*, and *P. cinerea*. White rot fungi are renowned for their ability to ensure complete lignocellulose degradation through the full decomposition of lignin structures (Qi et al., 2023). While *C. unicolor* and *T. pubescens* are recognized as ecologically and biotechnologically significant wood-degrading basidiomycetes with high lignocellulose-degrading abilities (Zhang et al., 2021; Abbas et al., 2024), *P. cinerea* is less commonly studied. Nonetheless, several studies suggest that this fungus may exhibit notable lignocellulolytic activity (Silvério et al., 2012).

*P. subcorticalis* and *C. pseudocladosporioides*, which previously have not been studied or described as lignocellulolytic enzyme producers, also demonstrated intense oxidation of the enzyme activity indicators. Based on the significant results observed during the screening study, these species were further studied to estimate enzyme production kinetics.

The quantitative study employing liquid media supplemented with Congo Red and Azure B confirmed that the fungi selected as the most potent to produce cellulolytic and ligninolytic enzymes in the initial screening studies indeed exhibited signs of enzyme secretion. Furthermore, the quantitative experiments underscore the role of enzymatic activity in the decolorization process. Previous studies have reported that the mechanisms of efficient decolorization involve both enzymatic degradation and absorption by fungal mycelium (Wang et al., 2017). In this study, the initial color change observed in both viable and inactivated fungal samples during cultivation in Congo Red or Azure B media also suggests potential dye adsorption into fungal mycelia. Nevertheless, enzymatic degradation was determined as the major factor in dye removal, considering the extremely significant alterations in both dye removal efficiencies between active and inactivated fungal cultures. Inactivated (autoclaved, no enzyme production detected) fungal mycelium showed on average less than 5% reduction in color intensity after 336 hours, emphasizing the importance of viable and enzymatically active fungi in the decolorization process.

Due to the ability to oxidize all three indicator substrates and significantly reduce Congo Red and Azure B coloration intensity by over 85%, three commercially obtained white rot fungi and three environmental isolates emerged as potential active producers of diverse lignocellulolytic enzymes (**Table 1**). Exhibiting both hydrolyzing and oxidative enzyme production and being white rot fungi, *B. adusta*, *T. versicolor*, *T. pubescens*, *C. unicolor*, and *P. cinerea* stand out as promising candidates for comprehensive lignocellulose degradation. Additionally, with its exceptional decolorization efficiency for Congo Red (98.66 ± 0.14%) and Azure B (99.78 ± 0.11%) and detected both cellulase and laccase activity, *C. pseudocladosporioides* can also be used in further investigation for its potential in lignocellulolytic enzyme production and application across biomass conversion, waste and wastewater treatment, and related fields reliant on lignocellulose-degrading enzymes (Sá et al., 2022; Sutaoney et al., 2024).

**Table 1:** Potential biotechnological applications for studied wood-decaying fungi.

Source	Fungal species	Characteristics	Potential applications
Cellulolytic fungi			
Commercially obtained	*Irpex lacteus*	White rot	Biofuel and bioenergy production, paper industry, textile industry, food and beverage processing, waste and wastewater treatment, cellulolytic enzyme production
Environmental isolate	*Fomitopsis pinicola*	Brown rot	
Ligninolytic fungi			
Commercially obtained	*Pleurotus dryinus*	White rot	Bioremediation, lignin pretreatment, biofuel and bioenergy production, textile industry, pharmaceutical industry, waste and wastewater treatment, ligninolytic enzyme production
Commercially obtained	*Pleurotus ostreatus*	White rot	
Environmental isolate	*Phacidium subcorticalis*	Saprophyte	
Lignocellulolytic fungi			
Commercially obtained	*Bjerkandera adusta*	White rot	Biomass conversion, environmental remediation, biofuel and bioenergy production, paper industry, textile industry, food and beverage processing, waste and wastewater treatment, lignocellulolytic enzyme production
Commercially obtained	*Trametes versicolor*	White rot	
Commercially obtained	*Pycnoporus cinnabarinus*	White rot	
Environmental isolate	*Trametes pubescens*	White rot	
Environmental isolate	*Cerrena unicolor*	White rot	
Environmental isolate	*Peniophora cinerea*	White rot	
Environmental isolate	*Cladosporium pseudocladosporioides*	Saprophyte	
Fungi with lower enzymatic efficiency			
Environmental isolate	*Trichoderma atrobrunneum*	Saprophyte	
Environmental isolate	*Trichoderma paraviridescens*	Saprophyte	
Environmental isolate	*Fusarium graminearum*	Plant pathogen	
Environmental isolate	*Trichoderma polysporum*	Saprophyte	

*T. versicolor* has already been identified as an effective producer of lignocellulose-degrading enzymes with promising prospects in lignocellulose-based bioeconomy (Tišma et al., 2021). The obtained results also align with the reported ability of *T. versicolor* to degrade Azure B through the enzymatic activities of MnPs and laccases, highlighting the potential of *T. versicolor* and other white rot fungi in the treatment of complex polluted wastewater (Zhang et al., 2023). Moreover, Zhang et al. (2022) reported the dye decolorization ability of extracellular ligninolytic enzymes produced from *C. unicolor* isolated from the decayed wood in Singapore, proving the applicability of lignocellulolytic white rot fungi in textile industries and textile wastewater decolorization. Based on the obtained results, *P. dryinus* and *P. ostreatus* were determined to be mostly ligninolytic fungi. While the observed cellulose-degrading abilities were relatively low compared to other species, the ability to decolorize Azure B and the exhibited laccase activity of up to 15,000 Units/L emphasized their potential for producing highly active laccases and peroxidases. These enzymes make them promising candidates for various applications. These include lignin pretreatment to enhance biomass conversion efficiency (Atiwesh et al., 2021) and pharmaceutical industry for the synthesis of several products like antioxidants and hormone derivatives (Senthivelan et al., 2016). Furthermore, fungal enzymes can play a valuable role in waste and wastewater treatment. Fungal laccases and peroxidases have been reported to degrade emerging contaminants, such as pharmaceutical residues and industrial pollutants (Sá et al., 2022; Dong et al., 2023).

Despite the well-known ability of white rot fungi to degrade lignocellulose, including lignin, in this study, *I. lacteus* demonstrated primarily cellulose-degrading capabilities compared to other white rot fungi like *B. adusta*, *T. pubescens*, and *C. unicolor*. Therefore, along with the brown rot fungus identified as *F. pinicola*, these species can be utilized in applications, such as cellulose-containing waste degradation, paper industry processes, and the production of cellulose-derived products like biofuels and high-value chemicals (Kuhad et al., 2011; Sutaoney et al., 2024). For instance, the ability of *I. lacteus* to secrete active cellulolytic enzymes has been previously utilized to produce efficient fermentable carbohydrate-releasing enzyme cocktails for lignocellulosic biomass enzymatic hydrolysis (Mezule and Civzele, 2020).

The results of this study demonstrate the remarkable potential of environmental isolates. In qualitative screening tests, while affecting a similar or smaller solid medium area, these fungi demonstrated more rapid and intensive decolorization of Congo Red, Azure B, and ABTS oxidation compared to commercially available cultures. These observations were also validated during the experiments in liquid media, where the isolated fungi, such as *T. pubescens*, *F. pinicola*, *C. unicolor*, *P. cinerea*, and *C. pseudocladosporioides* demonstrated a high potential to secrete active cellulose-degrading enzymes, and *T. pubescens*, *C. pseudocladosporioides*, and *C. unicolor* ensured nearly complete Azure B decolorization. Moreover, environmental isolates like *T. pubescens* and *P. cinerea* showed competitive performance against Congo Red with decolorization rates of 1.23 ± 0.01% per hour and 1.22 ± 0.01% per hour within the first 96 hours, comparable to commercial strains like *I. lacteus* (1.16% per hour), highlighting their potential as novel bioremediation agents. Similarly, the environmental isolates *T. pubescens* and *C. pseudocladosporioides*, demonstrated rapid Azure B degradation rates of 1.25% per hour and 1.70% per hour as the initial response.

Some environmental isolates also showed more superior activity of both cellulolytic and ligninolytic enzymes. The isolated culture of *F. pinicola* showed exceptional capability to produce highly active cellulose-degrading enzymes that was 6.3 times higher (*p* < 0.05) than the activity of *I. lacteus*, the most effective cellulolytic enzyme producer among the commercial cultures tested. *T. pubescens* and *C. unicolor* also demonstrated notable cellulase producing capabilities comparable to *I. lacteus* and more superior than other studied commercial species, such as *T. versicolor* and *B. adusta*. Moreover, the most exceptional lignin-degrading potential was also demonstrated by the fungus isolated from the environment. The isolated *T. pubescens* stands out with the laccase activity more than 200,000 Units/L, which is extremely significant difference compared to other studied fungi (*p* < 0.05). This study also demonstrates the potential of less studied *P. cinerea* and previously undescribed lignocellulolytic fungal species, such as *P. subcorticalis* and *C. pseudocladosporioides*, which, along with previously studied *I. lacteus* (Qin et al., 2018; Mezule and Civzele, 2020), *B. adusta* (Horisawa et al., 2019; Ilić et al., 2024), *T. versicolor* (Tišma et al., 2021), and *P. dryinus* (Elisashvili et al., 2006; Civzele et al., 2023), efficiently degraded indicator substrates, revealing them as suitable for lignocellulosic material treatment and other lignocellulose, cellulose, or lignin-related processes.

Thus, the obtained environmental fungal isolates from temperate forests can be regarded of high biotechnological importance due to their specific adaptation to the environmental conditions and consequently more active enzyme production capability. However, to fully assess and understand the potential of these strains, thorough enzyme gene profiling must be performed. Moreover, since other ligninolytic enzymes such as manganese peroxidase (MnP) and lignin peroxidase (LiP) play important roles in lignin degradation alongside laccase (Andlar et al., 2018), further studies can explore the activity of these enzymes to gain a more comprehensive understanding of the fungal ligninolytic system.

The observed ability to degrade Congo Red and Azure B also broadens the application of these fungi in the removal of the artificial dyes. Congo red is an azo dye applied in many industries like textiles, cosmetics, pigment, leather, food, pharmaceuticals, pulp, and paper; however, its extensive use leads to industrial pollution in the environment (Siddiqui et al., 2023). Azure B dye is also traditionally and widely used in the textile industry (Haq et al., 2018). Since the majority of the dyes have complex chemical structures, as well as enhanced stability, their degradation and removal from wastewaters is a challenging issue (Forgacs et al., 2004; Morsy et al., 2020). The study revealed the capabilities of *F. pinicola*, *P. cinerea*, *T. pubescens*, *C. unicolor*, and *C. pseudocladosporioides* to reduce the Congo Red-induced color intensity by more than 95% and the ability of *T. pubescens*, *C. unicolor*, and *C. pseudocladosporioides* to ensure almost complete Azure B removal from the liquid media. Thus, these environmental isolates possess a high potential to be a promising sustainable and environmentally friendly tool for dye-industry wastewater treatment and environmental remediation. To better confirm the bioremediation potential of these fungi, testing a broader range of dyes is essential. Therefore, investigation into these fungal species isolates should be continued to further expand their enzymatic activity profiles, optimize cultivation conditions, and evaluate their broader applications.

## 5 Conclusions

Environmental isolates from boreal coniferous and nemoral summer green deciduous forests, particularly *T. pubescens*, *C. unicolor*, and *C. pseudocladosporioides*, displayed remarkable efficiency in degrading Congo Red and Azure B, achieving over 98% removal of Congo Red and up to 99.80% decolorization of Azure B within 336 hours. Laccase production in *T. pubescens* exceeded 208 U/mL, significantly surpassing the levels observed in both commercial and other environmental fungal strains. The study also demonstrated a high potential for cellulolytic and ligninolytic enzyme production observed in lesser-studied and previously undescribed species, such as *P. cinerea*, *P. subcorticalis*, and *C. pseudocladosporioides*. These environmental isolates, together with active enzyme-producing white rot fungi like *B. adusta*, *T. versicolor*, and *I. lacteus*, have the potential not only in lignocellulose biomass conversion but also proved to be efficient candidates for complex chemical removal in waste and wastewater treatment, particularly in the textile industry. Furthermore, the findings of this study will support the set-up of new lignocellulolytic enzyme producers to advance in-house enzyme production systems in temperate climatic zones. The results can also serve as a foundation for the in-depth characterization of novel enzyme mechanisms in fungi previously not related to lignocellulolytic enzyme production.

## Data Availability

The original contributions presented in the study are included in the article/supplementary material. Further inquiries can be directed to the corresponding author.
